# Implementation of evidence-informed practice through central network actors; a case study of three public health units in Canada

**DOI:** 10.1186/s12913-017-2147-x

**Published:** 2017-03-15

**Authors:** Reza Yousefi Nooraie, Alexandra Marin, Robert Hanneman, Lynne Lohfeld, Maureen Dobbins

**Affiliations:** 1 Institute of Health Policy, Management, and Evaluation, University of Toronto, Toronto, Canada; 2 Department of Sociology, University of Toronto, Toronto, Canada; 3 Department of Sociology, College of Humanities, Arts, and Social Sciences, University of California, Riverside, USA; 4 Queen’s University Belfast, Centre for Public Health, Belfast, UK; 5 School of Nursing and Department of Health Research Methods, Evidence and Impact, McMaster University, Hamilton, Canada; 6 175 Longwood Road South, Suite 210a, Hamilton, ON L8P 0A1 Canada

**Keywords:** Evidence-informed decision-making, Social network analysis, Centrality, Local opinion leaders

## Abstract

**Background:**

Workforce development is an important aspect of evidence-informed decision making (EIDM) interventions. The social position of individuals in formal and informal social networks, and the relevance of formal roles in relation to EIDM are important factors identifying key EIDM players in public health organizations. We assessed the role of central actors in information sharing networks in promoting the adoption of EIDM by the staff of three public health units in Canada, over a two-year period during which an organization-wide intervention was implemented.

**Methods:**

A multi-faceted and tailored intervention to train select staff applying research evidence in practice was implemented in three public health units in Canada from 2011 to 2013. Staff (*n* = 572) were asked to identify those in the health unit whom they turned to get help using research in practice, whom they considered as experts in EIDM, and friends. We developed multi-level linear regression models to predict the change in EIDM behavior scores predicted by being connected to peers who were central in networks and were engaged in the intervention.

**Results:**

Only the group of highly engaged central actors who were connected to each other, and the staff who were not engaged in the intervention but were connected to highly engaged central actors significantly improved their EIDM behavior scores. Among the latter group, the staff who were also friends with their information sources showed a larger improvement in EIDM behavior.

**Conclusions:**

If engaged, central network actors use their formal and informal connections to promote EIDM. Central actors themselves are more likely to adopt EIDM if they communicate with each other. These social communications should be reinforced and supported through the implementation of training interventions as a means to promoting EIDM.

## Background

A crucial yet less developed aspect of evidence-informed decision making (EIDM) in public health is workforce development [1]. Training EIDM experts is an important consideration in administrative EIDM, since the majority of public health workers lack formal training and expertise to deal with the research evidence [2, 3]. EIDM is relevant to a broad spectrum of organizational roles ranging from front-line service providers to senior management, and a single level competency in EIDM does not reflect the diversity of public health decisions [4]. Consequently, a crucial step in designing interventions to promote EIDM is engaging the appropriate target players [5–8].

Individuals live in social networks in which they constantly interact with other members. ‘Centrality’ of individuals is one of the basic structural measures of social networks to identify prominent actors who are involved in many network relations [9]. Central actors have better access to resources and support as a result of their advantageous position in the communication network [10]. They also exert stronger influence on their environment due to their multitude of ties and the position of trust and power they may have as a result of their network position [11]. Consequently, identifying central actors in social networks is a valid and reliable technique to finding “opinion leaders” (OL) [12], who are individuals nominated by their peers as being influential [13] and able to informally affect others’ attitudes and behaviours in a desired way [14]. This position is not part of the formal role of people in an organization, but is ascribed to people as a result of their competence, accessibility, trustworthiness, and conformity to social norms [12]. Several studies in various disciplines have used network analysis approaches to identify OLs and train them to promote behavior change, including smoking cessation and HIV prevention [15, 16]. Flodgren et al. conducted a systematic review on the effect of OLs, and found that OLs, alone or in combination with other interventions, might successfully promote evidence-based practice, which was comparable to other known interventions, but effectiveness varied between and within studies [17]. However, due to the heterogeneous nature of the studies, the authors were not able to suggest the best way to identify OLs, involve them in the intervention, or what the optimal contextual environment was for OLs to have an impact. There is little agreement among various methods of identifying OLs [18, 19], due to the fact that different methods emphasize different aspects of opinion leadership. In addition, randomized controlled trial designs can only assess the average effect of OLs on the study sample, and generally neglect the fact that the social influence happens through social networks and its effects are not randomly distributed in the population. It is important to learn more about the attributes of central actors in information sharing networks, and the usefulness of their engagement in EIDM interventions, and the mechanism by which they influence the behavior of their peers.

We studied the association between network structure and adoption of EIDM in three public health units in Ontario, Canada, where an organization-wide intervention was implemented to promote EIDM. We tested the effect of seeking information from knowledgeable and central peers in changing the staff’s behavior towards EIDM. We also assessed the additional role of friendship in the social influence of central actors.

### Theoretical framework

Socially powerful and effective individuals can modify the beliefs and behaviors of people through the process of social influence. Referent and expert power are two main presentations of social power [20], which are characterized in local OLs. In ambiguous situations individuals compare themselves with socially powerful individuals, in order to reduce mental conflicts [21]. So the ambiguity of the decisions is a critical factor that determines the role of social influence. Given the ambiguous nature of public health decisions and barriers to adoption of EIDM by health practitioners [22], we expected that the social influence would affect the adoption of EIDM by staff in public health departments.

In this study, a subgroup of the staff of three public health units participated in trainings and work groups to promote EIDM. We hypothesized that the Staff who were engaged in the EIDM intervention would influence the adoption of EIDM by their peers (Hypothesis H1), as a result of the expert power they gained through the trainings.

However, not all engaged staff were expected to have similar social influence power. We hypothesized that the social influence of engaged staff would be higher if they were central in information-seeking networks. In other words, central actors who were engaged in the intervention would influence the adoption of EIDM by their peers (Hypothesis H2).

The other important foundation of social influence is the solidarity of relationships, and the role of trust and similarity [23]. According to social learning theory, people learn by observing the behavior of and non-verbal communication of their trusted peers [24]. The role of informal routes of connection sometimes is even more important than formal connections in changing behavior [25]. In order to assess the role of informal connections, and the extent to which central actors use their friendship ties to promote EIDM, we hypothesized that central actors who were engaged in the intervention would influence the adoption of EIDM by their friends to a larger extent than other peers (Hypothesis H3).

## Methods

### Study context

The health units varied in size, complexity, and commitment to EIDM. Units A and B mainly served large urban populations (>1.5 million population), and unit C served a smaller mixed urban–rural community (~600,000 population).

At the start of the study in 2011, A had a 10-year strategic plan to achieve EIDM and a specific budget line for individual capacity development activities to meet this goal, and assigned project specialists to practice-based teams, who were Masters level trained staff experienced in finding and interpreting research evidence, with responsibility for conducting literature reviews to address practice issues. Leadership at unit A strongly advocated EIDM, and was directly involved in the implementation of the intervention, monitored its progress, and promoted staff participation throughout the study. In units B and C engagement in the study was more division-based and less widespread. Unit B also identified EIDM as a strategic priority and attached health promotion consultants to specific teams to conduct literature reviews to address practice issues. At unit C responsibility for synthesizing evidence for practice issues rested with program managers and key front line staff. However this health unit did not have a strategic plan in place specifically for EIDM although it had dedicated some resources for capacity development.

### The intervention

The intervention was multi-faceted and tailored to meet the needs and characteristics of each health unit [26]. It was implemented during a 22 month period, with the aim of facilitating EIDM in the public health units. It included knowledge broker (KB) mentoring of small groups through the EIDM process to answer practice-relevant questions; one-day educational workshops; and one-to-one consultation and support by the KB [27]. The KB provided support on various steps of EIDM: formulating the questions, searching for evidence, appraising the scientific quality of the evidence, synthesizing the evidence, apprising the applicability and transferability of the findings, and applying it to local practice. The research group worked with the decision makers of three health units to tailor the content and organization of the intervention to the needs and priorities of each health unit.

We classified a group of staff as highly engaged in the intervention, based on information obtained from the KB’s journals where she tracked the teams she worked with, attendance lists from the large-group training sessions, and data exported from the online survey implemented at baseline and follow-up. In total, 84/1907 (4.4%) staff members were highly engaged in the intervention. In unit A, 53 staff members (8% of 638 total workforce) were identified as highly engaged in the intervention, and were involved in developing a total of 18 rapid evidence reviews [25]. In unit B, thirteen staff (1% of 1068 total workforce) were highly engaged in the intervention, and developed 5 rapid evidence reviews. At unit C, the number of highly engaged staff was 18 (9% of 201 total workforce), who developed 5 rapid evidence reviews.

### Data collection

Senior management of the three health units invited staff to participate in a confidential online survey at baseline and after the intervention (in 2011 and 2013). The invitation letters provided information on the purpose and methods of the study, the importance of their contribution, and the link to the online survey. However, the management was not aware of the identity of participating staff. The study was approved by the Institutional Review Board of McMaster University and ethics boards in each health unit. A written informed consent was obtained from the participants.

The online survey included demographic questions, EBP Implementation scale (comprised of 18 questions), and questions corresponding to the respondent’s social network. Respondents answered four randomly ordered name generator questions about their social relations. Respondents named staff members in the unit whose input they regularly sought to help them integrate research evidence into practice-based decisions (information seeking), who were experienced and knowledgeable in finding and applying research evidence (expertise recognition), and who they considered friends (friendship).

In order to assess the extent to which respondents implemented EIDM in their practice, the Evidence Based Practice (EBP) Implementation scale of Melnyk and colleagues [28] was administered. Respondents were asked to provide the frequency of their involvement in 18 evidence-based practice activities during the 8 weeks prior to the assessment, using a 5-point frequency scale. The EBP activities included different aspects of using and appraising evidence to inform public health practice, and sharing the evidence with colleagues and clients. This tool has good internal consistency (Chronbach’s alpha > 0.9), and showed a significant association with educational level and prior contact with EBP in other studies [28]. The scale was administered at baseline and follow up. Participants received two reminder emails one week apart to encourage higher response rates [29].

### Analysis

For each respondent we calculated the in-degrees (number of peers who identified that member as the information source or expert) and his/her number of friends.

We identified central actors based on their in-degree centrality, which is the frequency of nomination by other members of the community. We defined central actors as individuals whose in-degree centrality in both information-seeking and expertise recognition networks at baseline was in the highest quartile.

We developed multi-level linear regression models to predict EBP implementation scores. The random levels included individuals (in which individual assessments of EBP implementation scores at baseline and follow up were nested), and public health units in which the individuals were nested. The predictors of the EBP implementation scores were: being a central actor, being highly engaged in the intervention, seeking information from at least one highly engaged peer at baseline (addressing hypothesis H1), seeking information from at least one highly engaged central peer at baseline (addressing hypothesis H2), and each variable’s interactions with time of assessment (baseline = 0,follow up = 1).

We also developed a similar three-level regression model in the subgroup of staff who sought information from at least one highly engaged central peer. The EBP implementation score was the dependent variable, which was predicted by the following variables and their interaction with time of assessment: being a central actor, being highly engaged in the intervention, being friends with at least one highly engaged peer at baseline, being friends with at least one highly engaged central peer at baseline (addressing hypothesis H3). The analysis was carried out in STATA 12.1 [30].

## Results

### The characteristics of central actors

In total, 611 (32% of total workforce of three health units) answered baseline and 820 (43%) answered follow up surveys. In unit A, 207 (32% of 638 total workforce) and 256 (40%) staff answered to baseline and follow up surveys. In unit B, 309 (29% of 1068 workforce of participating organizational divisions) and 404 (38%) staff answered to baseline and follow up assessments. In unit C, 95 (47% of 201 workforce) and 160 (79%) staff answered to baseline and follow up surveys. Table 1 shows the baseline characteristics of central actors at each public health unit. In all three health units the central actors had an average of 3 more years of public health experience than the rest of staff. In all three units central actors were more educated than others. The frequency of managerial and EIDM professional roles (e.g. project specialist, or health promotion consultant, as defined by the health unit) in central actors was almost three times more than other staff in all three health units. However, 43% of the central actors at unit A (*n* = 12), 20% at unit B (*n* = 6), and 67% at unit C (*n* = 6) were not a manager or EIDM professional. This group included mainly supervisors (*n* = 5) and nutritionists (*n* = 3) in unit A, supervisors (*n* = 3) and program evaluators (*n* = 2) in unit B, and epidemiologists (*n* = 4) in unit C. The average baseline EBP implementation score of central actors was higher than the rest of the staff, which was statistically significant at units A and B. In all three health units, the central actors in information seeking and expertise networks were also more central than the others in friendship network.

**Table 1:** The characteristics of network actors in three public health units who responded to baseline and/or follow up surveys

		Unit A		Unit B		Unit C	
		Central (*n* = 28, 9%)	Others (*n* = 288)	Central (*n* = 31, 6%)	Others (*n* = 503)	Central (*n* = 9, 5%)	Others (*n* = 176)
Female (%)		23 (82%)	259 (90%)	26 (84%)	456 (91%)	8 (89%)	142 (81%)
Years of public health experience; mean (SD)		13 (9)*	10 (8)	17 (7)	14 (9)	15 (8)	12 (9)
Educational degree	Diploma	0	41 (14%)	0	67 (13%)	0	57 (32%)
	Baccalaureate	11 (39%)	173 (60%)	6 (19%)	259 (52%)	3 (33%)	104 (59%)
	Masters	16 (57%)	68 (24%)	21 (68%)	151 (30%)	6 (66%)	14 (8%)
	Doctorate	1 (4%)	5 (1.7%)	4 (13%)	16 (3%)	0	1 (0.5%)
Managerial	Highly engaged	4 (14%)	10 (3%)	0	1	3 (33%)	3 (2%)
	Not engaged	2 (7%)	12 (4%)	11 (35%)	48 (10%)	0	16 (9%)
EIDM professional	Highly engaged	8 (29%)	5 (2%)	3 (10%)	6 (1%)	-	-
	Not engaged	2 (7%)	9 (3%)	11 (35%)	77 (15%)	-	-
Other	Highly engaged	5 (18%)	21 (7%)	0	3 (0.5%)	2 (22%)	10 (6%)
	Not engaged	7 (25%)	230 (80%)	6 (19%)	463 (92%)	4 (44%)	144 (82%)
Baseline EBP implementation score mean (SE)		14.5 (7.4)**	9.5 (8.5)	13.5 (6.8)*	9.4 (10)	9.8 (7.3)	7.5 (7)
Degree in friendship network		3.6 (2.1)***	1.8 (1.4)	3 (1.7)**	2 (1.4)	3 (0.8)**	1.9 (1.1)

*:*p* < 0.05, **:*p* < 0.01, ***:*p* < 0.001

Central actors were defined as the staff who were at the 4^th^ quartile of centrality in information seeking and expertise recognition networks: Unit A (in-degree of 3+ in the information-seeking network and 1+ in the expertise-recognition), Unit B (in-degree of 3+ in the information-seeking network and 2+ in the expertise-recognition), unit C (in-degree of 2+ in the information-seeking network and 2+ in the expertise-recognition)

**Fig. 1:**
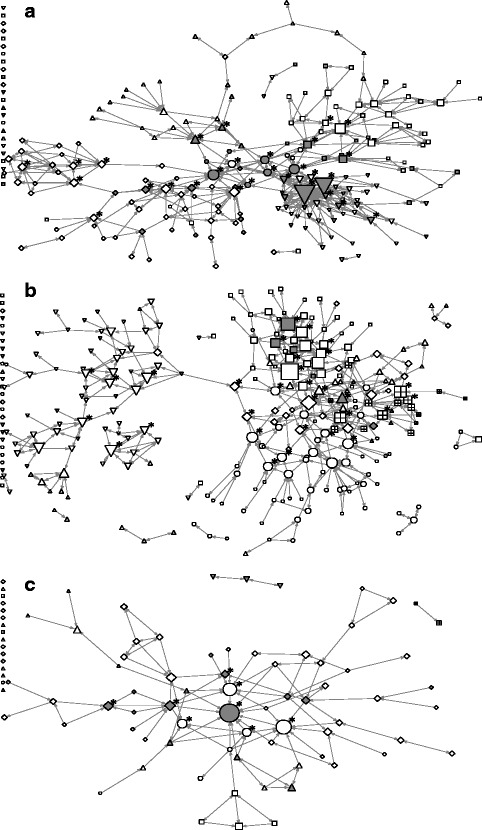
Information-seeking networks at baseline in three public health units. *Grey* nodes were highly engaged in the intervention. The shapes of the nodes represent organizational divisions. Node size is proportional to the in-degree centrality. Asterisks represent central network actors. **a** unit A, **b** unit B, **c** unit C

In total, 8% of respondents (*n* = 84) were highly engaged in the intervention, and a third of whom (*n* = 25) were central actors in social networks, with varying proportions in three health units. In unit A, 17% (*n* = 53) of respondents were highly engaged, who comprised of 61% (*n* = 17) of central actors. Apart from the two highly engaged central actors in one practice-based division, the majority of highly engaged central actors were in the supervisory/administrative division (Fig. 1a: circles). In unit B, only 2% (*n* = 13) of the staff were highly engaged. The central actors in this unit were distributed in various divisions (Fig. 1b), and only three of whom were engaged in the intervention (10%). In unit C, 10% (*n* = 18) of the staff engaged in the intervention, who comprised of 56% (*n* = 5) of central actors. Many central actors were epidemiologists in the supervisory/administrative division (Fig. 1c: circles), who were not engaged in the intervention.

About 60% of highly engaged central actors in the three units had a Masters degree or higher, which was significantly more than other groups. In unit A, highly engaged central actors were mainly managers and project specialists, in unit B all were health promotion consultants, and in unit C mainly management staff. Appendix 1 shows the characteristics of respondents to baseline and follow-up surveys. Appendix 2 shows the average scores of the items in EBP Implementation Scale questionnaire, before and after the intervention.

### Change in EBP implementation scores over time

Table 2 shows the coefficients of the regression models to predict EBP implementation scores at follow up, pooling all the data from three health units. Model A that only included being highly engaged in the intervention as the predictor showed a significant improvement in EBP scores from baseline in the group who were engaged in the intervention (mean increase: 2.4). Model B that also included being a central actor as the predictor showed that the highly engaged central staff had higher baseline EBP scores (diff from not engaged: 5.5), and also were the only group who significantly improved their EBP scores at follow up (mean increase: 4.2). Model C that also included information seeking behavior showed that two groups of staff significantly increased their EBP implementation scores over time: a group of highly engaged central actors who sought information from each other (mean increase: 4.9); and a group of staff who were not themselves highly engaged in the intervention but sought information from a highly engaged central actors (mean increase: 2.5), confirming hypothesis H2. Engagement in the intervention and seeking information from a highly engaged peer did not necessarily have a significant effect on EBP implementation scores, not confirming hypothesis H1.

**Table 2:** Regression models to predict EBP behavior scores over time in different groups of respondents based on their information seeking patterns

Regression coefficients		Model 1	Model 2	Model 3
Respondents	Seeking information from a peer who is:			
Baseline				
Not highly engaged	Not highly engaged	9.1(0.7)	9.1(0.7)	10.5(0.5)
	Highly engaged, but not central			−3.2(1.4)*
	Highly engaged central			−0.7(1.1)
Highly engaged, but not central	Not highly engaged	1.6(1.1)	−0.3(1.4)	1.5(2.3)
	Highly engaged, but not central			−4.3(3.0)
	Highly engaged central			−2.1(1.9)
Highly engaged central	Not highly engaged		5.5(1.9)**	5.5(5.2)
	Highly engaged, but not central			2.0(4.8)
	Highly engaged central			4.3(2.1)*
Change from baseline				
Not highly engaged	Not highly engaged	0.07(0.4)	0.07(0.4)	−0.8(0.6)
	Highly engaged, but not central			−0.3(1.3)
	Highly engaged central			2.5(1.1)*
Highly engaged, but not central	Not highly engaged	2.4(1.0)*	1.4(1.3)	−0.5(2.3)
	Highly engaged, but not central			−1.8(3.2)
	Highly engaged central			2.9(1.7)
Highly engaged central	Not highly engaged		4.2(1.8)**	−0.3(4.7)
	Highly engaged, but not central			4.8(7.5)
	Highly engaged central			4.9(1.9)**
Random effects variance				
Health Units		1.03(1.1)	1.0(1.0)	~0
Individuals		52.5(4.3)	51.7(4.3)	47(4.9)
Residual		36.1(2.7)	36.0(2.7)	33(2.9)

1: The coefficients for all levels at baseline represent the difference from the first row

2: The coefficients for all levels at ‘change from the baseline’ represent the difference from their baseline

*:*p* < 0.05, **:*p* < 0.01

The pooled analysis of the effect of friendship network on changes in EBP implementation scores in the group of staff who sought information from highly engaged central actors (Table 3), showed that three groups of staff significantly improved their behavior: the staff who were not highly engaged but had at least one highly engaged central peer in their friends list (mean increase: 3.3), non central highly engaged group who had at least one highly engaged central peer in their friends list (mean increase: 5.7), and highly engaged central actors regardless of their friendship status. These findings confirmed hypothesis H3.

**Table 3:** Regression model to predict the EBP behavior scores over time based on their friendship patterns in the subgroup who sought information from highly engaged central staff

		Regression coefficients
Respondents	Friends with someone who is:	
Baseline		
Not highly engaged	Not highly engaged	9.9(1.2)
	Highly engaged, but not central	−3.5(2.1)
	Highly engaged central	−0.8(1.7)
Highly engaged, but not central	Not highly engaged	0.1(3.4)
	Highly engaged, but not central	−3.3(2.4)
	Highly engaged central	−1.8(2.7)
Highly engaged central	Not highly engaged	6.1(3.2)
	Highly engaged, but not central	0.4(4.3)
	Highly engaged central	5.6(2.4)*
Change from baseline		
Not highly engaged	Not highly engaged	−0.7(1.1)
	Highly engaged, but not central	2.3(1.8)
	Highly engaged central	3.3(1.4)*
Highly engaged, but not central	Not highly engaged	−2.0(3.2)
	Highly engaged, but not central	2.6(2.3)
	Highly engaged central	5.7(2.3)*
Highly engaged central	Not highly engaged	8.1(3.2)*
	Highly engaged, but not central	3.0(3.8)
	Highly engaged central	4.8(2.0)*
Random effects variance		
Health Units		0.6(1.5)
Individuals		29.2(5.4)
Residual		21.8(3.0)

1: The coefficients for all levels at baseline represent the difference from the first row

2: The coefficients for all levels at ‘change from the baseline’ represent the difference from their baseline

*:*p* < 0.05

## Discussion

Our findings showed that staff who were connected to highly engaged central actors showed significant improvement in their EIDM behavior, even if they were not themselves highly engaged in the intervention. Among staff who were connected to highly engaged central actors, friendship ties with highly engaged central peers significantly improved EIDM behavior. Highly engaged central staff themselves were more likely to improve their EIDM behavior if they were connected to other highly engaged staff. These findings highlight the role of central actors in information seeking networks as opinion leaders who can influence the EIDM behavior of their peers, and also the importance of connectedness among opinion leaders as a facilitator of their own behavior change.

### Social influence of central actors

Individuals may influence each other through various routes, which mainly rest on hierarchy (social power) and solidarity [23]. These may establish two forms of trust: cognitive (based on the individual’s belief about peers reliability and competence) and affective (based on emotional bonds) [31], through which people influence others. Being recognized as an expert provides individuals with social power through the direct perception of expertise (expert power) and also being referred and recognized by many as experts (referent power) [20].

Social influence can also work through affective connections. Individuals are influenced by the behaviour of their friends and peers with whom they share values and interests [23]. Friendship is the result of shared values and frequent communication, and happens in the context of mutual trust [32]. Friendship ties are more stable than formal advice-seeking connections [33], and provide a safe foundation for social influence, especially in risky situations [34]. In our study, friendship ties reinforced behavior change among not-engaged staff. This subgroup probably differed considerably from highly engaged staff in terms of the value of EIDM in their practice and their position in the organization. So we can assume that for this subgroup changing behavior towards EIDM was probably more risky and less convenient, compared to staff who were recognized and chosen by management to get engaged in the intervention.

Our findings showed that central actors in information seeking and expertise networks fulfill the conditions of opinion leadership because of their prominent formal and informal ties and significant social influence. Central network actors have better access to resources and are more likely to be aware of new opportunities (such as EIDM training in this study) [35]. In addition, because of their central position they are more likely to engage in risky behaviors and adopt innovations [11, 14]. Their influence on their peers’ behavior, and their motivation to try innovations make the central network actors suitable people to engage in organizational interventions [17, 36]. Consequently, we recommend identifying and engaging central network actors in EIDM interventions [12, 36].

We used a sociometric approach to identify OLs. Different techniques to define OLs lead to the identification of different subgroups [18]. Each technique emphasizes on different aspects of the complex construct of opinion leadership. For example, a self-identification technique may select OLs based on their values and traits, whereas a sociometric approach emphasizes the practical usefulness of the OLs from the perspective of their peers [12]. Therefore it is suggested that different techniques should be combined to identify those who meet a greater number of criteria for opinion leadership [12]. In our study we used a combined approach, defining OLs as staff members who were at the fourth (highest) quartile of both the information-seeking and expertise- recognition networks through which we selected participating OLs based on the conceptual overlap between these two networks. When using sociometric techniques to identify OLs, this combined approach seems more justifiable, because it captures the group who fulfill more than one leadership characteristic. The significant social influence effect of this subgroup in our quantitative analysis implies that our identification technique was also empirically sensitive in identifying the channels of social influence in public health units.

### Behavior change of central actors

We found that central actors highly engaged in the intervention were more likely to adopt EIDM if they were connected to other highly engaged central peers. Although OLs are sometimes considered as innovative and creative [37, 38], they generally tend to conform to social norms [14] and are, in fact, more often conservative and behave within the normative bounds of their social networks. They often monitor the climate and advocate change when the advantages of the innovations are apparent or the change in norms is inevitable [12]. OLs may impede the dissemination and implementation of interventions that they consider risky and radical [39]. So depending on the readiness of the organization to adopt change and the risk of adopting innovations, OLs may act to promote or oppose the change [14].

In addition, the association between OLs and their peers is not a unidirectional connection between a leader and followers. OLs are themselves influenced by their peers and may change their behavior to conform to group or organization-wide norms [19, 40].

The significant behavior change in central actors in this study shows a higher motivation and readiness in this group, which is probably affected by their social position as experts in EIDM. In addition, the behavior change in a subgroup of highly engaged staff could be the result of communication and support in small groups through the course of the study. We conducted a qualitative study to understand the process of behavior change among highly engaged central network actors [41]. We learned that, if supported by the health unit leadership, highly engaged staff formed closely connected clusters through which they shared their concerns and progress stories [42]. These clusters consisting of individuals with similar expertise, interests, and challenges who help each other through communication and feedback resembles communities of practice [43, 44]. Interactions in small groups and the influence that people have on each other assist in the formation of shared understanding and agreements, and subsequently evolving social norms [45], which subsequently motivates the more conservative central actors to promote EIDM. Formation of network ties among highly engaged staff or strengthening already existing connections are network alteration techniques, which are among theoretically effective but less studied network interventions [46].

### Strengths and limitations

This study is unique in its assessment of the effect of social relations in adoption of EIDM in public health. Our study explored the construct of opinion leadership beyond assessing the overall effectiveness of OL, and provided longitudinal evidence on the process of their influence.

However, the findings of this case study only provide clues to potential organizational complexities that should ideally be studied in a more systematic way. Even though we found some evidence that the behavior change in staff is beyond the effect of common context, a longitudinal network analysis that takes direct dyadic relations into account and controls for the effect of social selection and common context is a more systematic analysis approach [47, 48]. In addition, controlled studies are needed to assess the effect of engagement of OLs as a step in tailoring EIDM interventions in public health organizations. Finally, the response rate in three health units, and the number of highly engaged staff in units B and C was small. As a result, the results of the regression models are more affected by the findings at unit A.

## Conclusions

The centrality in information and expertise networks coincided with a key position in friendship networks and also higher EIDM behavior scores. If engaged, central actors used their formal and informal connections to promote EIDM in their social networks. We recommend that identifying central actors in social networks and engaging them in workforce development interventions should be incorporated into the tailoring process when developing programs to promote EIDM in public health. In addition, forming and sustaining connections among central actors to reinforce and support each other throughout the process of behavior change should be considered as part of the implementation strategies.

## Appendix 1

**Table 4:** The baseline characteristics of the respondents to baseline and follow up surveys

		Unit A		Unit B		Unit C	
		Baseline	Follow up	Baseline	Follow up	Baseline	Follow up
Female (%)		186 (90%)	226 (88%)	283 (92%)	359 (89%)	75 (79%)	130 (81%)
Years of public health experience; mean (SD)		12 (8)	10 (8)	16 (9)	14 (9)	13 (9)	12 (9)
Educational degree	Diploma	25 (12%)	34 (13%)	23 (8%)	55 (14%)	19 (20%)	54 (34%)
	Baccalaureate	122 (52%)	149 (58%)	153 (50%)	198 (49%)	63 (66%)	85 (53%)
	Masters	57 (28%)	68 (27%)	122 (39%)	125 (31%)	12 (12%)	18 (11%)
	Doctorate	2 (1%)	5 (2%)	11 (4%)	13 (3%)	1 (1%)	1 (1%)
Job titles	Managerial	23 (11%)	25 (10%)	51 (17%)	47 (12%)	17 (18%)	17 (11%)
	EIDM professional	33 (16%)	38 (15%)	76 (25%)	72 (18%)	-	-
Highly engaged		48 (23%)	44 (17%)	13 (4%)	13 (3%)	18 (19%)	13 (8%)

## Appendix 2

**Table 5:** The average (SD) frequency that the items in EBP Implementation in Public Health Scale applied to the respondents in the past 8 weeks, on a 5-point scale [28]. The average scores of the items in EBP Implementation Scale questionnaire, before and after the intervention

	Baseline	Follow up
Used evidence to change my public health practice	1 (1.1)	1 (1)
Critically appraised evidence from a research study	0.8 (1)	0.7 (1)
Generated a PICO question about my public health practice	0.2 (0.6)	0.3 (0.6)
Informally discussed evidence from a research study with a colleague	1.4 (1.1)	1.3 (1.2)
Collected data on a patient problem	0.6 (1.2)	0.7 (1.2)
Shared evidence from a study or studies in the form of a report or presentation to more than 2 colleagues	0.6 (0.9)	0.6 (0.9)
Evaluated the outcomes of a practice change	0.3 (0.7)	0.3 (0.7)
Shared an evidence based guideline with a colleague	0.7 (0.9)	0.7 (0.9)
Shared evidence from research with a client	0.6 (1.1)	0.7 (1.2)
Shared evidence from a research study with a multi-disciplinary team member	0.6 (0.9)	0.6 (0.9)
Read and critically appraised a clinical research study	0.4 (0.8)	0.5 (0.9)
Accessed the Cochrane database of systematic reviews	0.3 (0.7)	0.4 (0.9)
Accessed the National Guidelines Clearinghouse	0.1 (0.4)	0.2 (0.6)
Used an evidence based guideline or systematic review to change public health practice where I work	0.4 (0.7)	0.4 (0.7)
Evaluated a care initiative by collecting client outcome data	0.2 (0.6)	0.3 (0.7)
Shared the outcome data collected with colleagues	0.3 (0.7)	0.3 (0.8)
Changed practice based on client outcome data	0.2 (0.6)	0.2 (0.6)
Promoted the use of evidence in practice to my colleagues	1 (1)	1 (1.1)

## Abbreviations

EBP: Evidence based practice
EIDM: Evidence-informed decision making
KB: Knowledge broker
OL: Opinion leader

## References

[1] Brownson R, Reis R, Allen P, Duggan K, Fields R, Stamatakis K, Erwin P (2014). Understanding administrative evidence-based practices: findings from a survey of local health department leaders. Am J Prev Med.

[2] Brownson R, Allen P, Duggan K, Stamatakis K, Erwin P (2012). Fostering more-effective public health by identifying administrative evidence-based practices: a review of the literature. Am J Prev Med.

[3] Orton L, Lloyd-Williams F, Taylor-Robinson D, O’Flaherty M, Capewell S (2011). The use of research evidence in public health decision making processes: systematic review. PLoS One.

[4] Maxwell M, Adily A, Ward J (2007). Promoting evidence-based practice in population health at the local level: a case study in workforce capacity development. Aust Heal Rev.

[5] Légaré F, Ratté S, Gravel K, Graham ID (2008). Barriers and facilitators to implementing shared decision-making in clinical practice: update of a systematic review of health professionals’ perceptions. Patient Educ Couns.

[6] Oxman A, Thomson M, Davis D, Hayes J (1995). No magic bullets: a systematic review of 102 trials of interventions to improve professional practice. CMAJ.

[7] Dobbins M, Davies B, Danseco E, Edwards N, Virani T (2005). Changing nursing practice: evaluating the usefulness of a best-practice guideline implementation toolkit. Nurs Leadersh (Tor Ont).

[8] Baker R, Camosso-Stefanovic J, Gilliss C, Shaw E, Cheater F, Flottorp S, Robertson N. Tailored interventions to overcome identified barriers to change: Effects on professional practice and health care outcomes. Cochrane Database Syst Rev. 2010;3:CD005470. 10.1002/14651858.CD005470.pub2 PMC4164371 20238340

[9] Wasserman S, Faust K (1994). Notation for social network data.

[10] Mehra A, Dixon AL, Robertson B (2006). The social network ties of group leaders: implications for group performance and leader reputation. Organ Sci.

[11] Ibarra H, Andrews S (1993). Power, social influence, and sense making: effects of network centrality and proximity on employee perceptions. Adm Sci Q.

[12] Valente T, Pumpuang P (2007). Identifying opinion leaders to promote behavior change. Heal Educ Behav.

[13] Majumdar S, Tsuyuki R, McAlister F (2007). Impact of opinion leader? endorsed evidence summaries on the quality of prescribing for patients with cardiovascular disease: a randomized controlled trial. Am Hear J.

[14] Rogers E (2003). Diffusion of innovations.

[15] Kelly J, Amirkhanian Y, Kabakchieva E, Vassileva S, McAuliffe T, DiFranceisco W (2006). Prevention of HIV and sexually transmitted diseases in high risk social networks of young Roma (Gypsy) men in Bulgaria: randomised controlled trial. BMJ.

[16] Valente T, Gallaher P, Mouttapa M (2004). Using social networks to understand and prevent substance use: a transdisciplinary perspective. Subst Use Misuse.

[17] Flodgren G, Parmelli E, Doumit G, Gattellari M, O’Brien M, Grimshaw J, Eccles M. Local opinion leaders: effects on professional practice and health care outcomes. Cochrane Database Syst Rev. 2011;8:CD000125. 10.1002/14651858.CD000125.pub4 PMC4172331 21833939

[18] Grimshaw JM, Eccles MP, Greener J, Maclennan G, Ibbotson T, Kahan JP, Sullivan F (2006). Is the involvement of opinion leaders in the implementation of research findings a feasible strategy?. Implement Sci.

[19] Iyengar R, Van den Bulte C, Valente TW (2011). Opinion leadership and social contagion in new product diffusion. Mark Sci.

[20] French J, Raven B, Cartwright D (1959). The bases of social power. Studies in social power.

[21] Erickson B. The relational basis of attitudes. In Social structures: A network approach. Edited by Wellman B, Berkowitz S. New York: Cambridge University Press; 1988:99–121.

[22] Graham I, Logan J, Harrison M, Straus S, Tetroe J, Craswell W, Robinson N (2006). Lost in knowledge translation: time for a map?. J Contin Educ Health Prof.

[23] Marsden PV, Friedkin NE (1993). Network studies of social influence. Sociol Methods Res.

[24] Bandura A (1977). Social learning theory.

[25] Yost J, Dobbins M, Traynor R, DeCorby K, Workentine S, Greco L (2014). Tools to support evidence-informed public health decision making. BMC Public Health.

[26] Dobbins M, Traynor R, Greco L (2015). Building capacity for evidence-informed decision making in Canadian public health. Implement Sci.

[27] Ciliska D, Thomas H, Buffett C (2012). An introduction to evidence-based public health and a compendium of critical appraisal tools for public health practice (revised).

[28] Mazurek Melnyk B, Fineout-Overholt E, Mays M (2008). The evidence-based practice beliefs and implementation scales: psychometric properties of two new instruments. Worldviews Evidence-Based Nurs.

[29] Dillman D (2007). Mail and internet surveys: the tailored design method.

[30] StataCorp (2011). Stata statistical software: release 12.

[31] McAllister D (1995). Affect-and cognition-based trust as foundations for interpersonal cooperation in organizations. Acad Manag J.

[32] Gibbons DE (2004). Friendship and advice networks in the context of changing professional values. Adm Sci Q.

[33] Lewis J, Weigert A (1985). Trust as a social reality. Soc Forces.

[34] Mayer R, Davis J, Schoorman F (1995). An integrative model of organizational trust. Acad Manag Rev.

[35] Wasserman S, Faust K (1994). Social network analysis: methods and applications.

[36] Valente T, Davis R (1999). Accelerating the diffusion of innovations using opinion leaders. Ann Am Acad.

[37] Childers T (1986). Assessment of the psychometric properties of an opinion leadership scale. J Mark Res.

[38] Van Eck P, Jager W, Leeflang P (2011). Opinion leaders’ role in innovation diffusion: a simulation study. J Prod Innov Manag.

[39] Gibson D (2005). Concurrency and commitment: network scheduling and its consequences for diffusion. J Math Sociol.

[40] Myers JH, Robertson TS (1972). Dimensions of opinion leadership. J Mark Res.

[41] Yousefi Nooraie R, Lohfeld L, Marin A, Hanneman R, & Dobbins M. Informing the implementation of evidence-informed decision making interventions using a social network analysis perspective; a mixed-methods study. BMC Health Services Research, 2017;17(1):122. 10.1186/s12913-017-2067-9 PMC5299784 28178958

[42] Yousefi-Nooraie R, Dobbins M, Marin A, Hanneman R, Lohfeld L (2015). The evolution of social networks through the implementation of evidence-informed decision-making interventions: a longitudinal analysis of three public health units in Canada. Implement Sci.

[43] Wenger E, McDermott R, Snyder W (2002). Cultivating communities of practice.

[44] Estabrooks C, Thompson D, Lovely J, Hofmeyer A (2006). A guide to knowledge translation theory. J Contin Educ Health Prof.

[45] Friedkin N, Johnsen E (1999). Social influence networks and opinion change. Adv Gr Process.

[46] Valente T (2012). Network interventions. Science (80-).

[47] Snijders TAB, Van de Bunt GG, Steglich CEG, van der Bunt G (2010). Introduction to stochastic actor-based models for network dynamics. Soc Networks.

[48] Steglich C, Snijders TAB, Pearson M (2010). Dynamic networks and behavior: separating selection from influence. Sociol Methodol.

